# Cognitive Change in Older Women Using a Computerised Battery: A Longitudinal Quantitative Genetic Twin Study

**DOI:** 10.1007/s10519-013-9612-z

**Published:** 2013-08-30

**Authors:** Claire J. Steves, Stephen H. D. Jackson, Tim D. Spector

**Affiliations:** 1 Department of Twin Research and Genetic Epidemiology, Kings College London, Block 7 South Wing, 3rd Floor, St Thomas’ Campus, Lambeth Palace Road, London, SE1 7EH UK; 2 Clinical Age Research Unit, Department of Clinical Gerontology, Kings College Hospital Foundation NHS Trust, London, UK

**Keywords:** Processing speed, Heritability, Cognition, Aging, Twin study, Accelerating change

## Abstract

Cognitive performance is known to change over age 45, especially processing speed. Studies to date indicate that change in performance with ageing is largely environmentally mediated, with little contribution from genetics. We estimated the heritability of a longitudinal battery of computerised cognitive tests including speed measures, using a classical twin design. 324 (127 MZ, 197 DZ) female twins, aged 43–73 at baseline testing, were followed-up after 10 years, using seven measures of the Cambridge Automated Neuropsychological Test battery, four of which were measures of response latency (speed). Results were analysed using univariate and bivariate structural equation modelling. Heritability of longitudinal change was found in 5 of the 7 tests, ranging from 21 to 41 %. The genetic aetiology was remarkably stable. The first principle component of change was strongly associated with age (*p* < 0.001) and heritable at 47 % (27–62 %). While estimates for heritability increased in all measures over time compared to baseline, these increases were statistically non-significant. This computerised battery showed significant heritability of age-related change in cognition. Focus on this form of change may aid the search for genetic pathways involved in normal and pre-morbid cognitive ageing.

**Electronic supplementary material:**

The online version of this article (doi:10.1007/s10519-013-9612-z) contains supplementary material, which is available to authorized users.

Over the age of 60 normal healthy adults start to show changes in cognition. Many cognitive functions, which have been slowly improving up to this point, start to plateau, and then decline (Schaie et al. 2004). These changes occur at the same time in life as a number of other challenges and stressors, for example changes in employment and increasing physical health problems. Whether cause or effect, these changes may have significant impact on quality of life despite not constituting dementia. Research into this area is timely as this population is swelling rapidly, the ‘baby boomers’ having started to turn 65 in 2011 (Daffner 2010). Populations show increasing variability in cognitive performance as they age, suggesting that there are factors that enable some individuals to preserve their functions better than others (McDaniel et al. 2007).

Twin studies can investigate the extent to which variation in a population is due to genes or environment. To date, there is sparce evidence that intra-individual differences in cognitive change with age are genetically determined. This stands in contrast to cognitive performance at a single time point, which is highly heritable (Lee et al. 2010; McArdle and Plassman 2009; McGue and Christensen 2002; Reynolds et al. 2005). Evidence for a genetic contribution to change with age includes heritability of brain morphology changes over a four-year follow up in older males (Pfefferbaum et al. 2004). In addition, using growth curve models, Reynolds et al. have shown that there is heritability of quadratic change in many cognitive tests (Reynolds et al. 2005). There is some evidence that such intra-individual quadratic change is more important than linear change in predicting conversion to dementia states (Small and Backman 2007). Intriguingly, McArdle and Plassman, investigating word recall longitudinally, found a genetic contribution to rate of change before age 74, but predominantly environmental factors driving change after this age (McArdle and Plassman 2009), a finding in common with some other systems in ageing (Steves et al. 2012).

It is well established that processing speed and reaction times are most sensitive to ageing within a normal population (Salthouse 2010). Computerized tests of speed of inspection, decision, and reaction are now possible, yet so far there have been no published longitudinal quantitative genetic studies of reaction time, and only one cross sectional study (Finkel and McGue 2007).

We aimed to describe the quantitative genetics of a battery of computerised tests which include a number of speed measures, including reaction time, choice reaction time, correct inspection time and correct decision time. Our hypothesis was that we would find significant heritability of cognitive ageing with a battery rich in such speed measures.

## Methods

### Study participants

This study utilised a subset population of the Twins UK registry of female volunteer twins who were recruited in media campaigns from 1992 onwards. The initial campaigns focused on diseases affecting older women predominantly, so the cohort in 1999 consisted of 96 % women, mainly in mid-life to old age. Because of this, and due to the fact that age-related quantitative traits may have different aetiologies in men and women (Weiss et al. 2006), only women were recruited into this study, which is part of the Healthy Aging Twin Study (Moayyeri et al. 2012; Spector and Williams 2006). Ethical approval for the present study was obtained from Guys and St Thomas’ Research Ethics Committee.

**Fig. 1:**
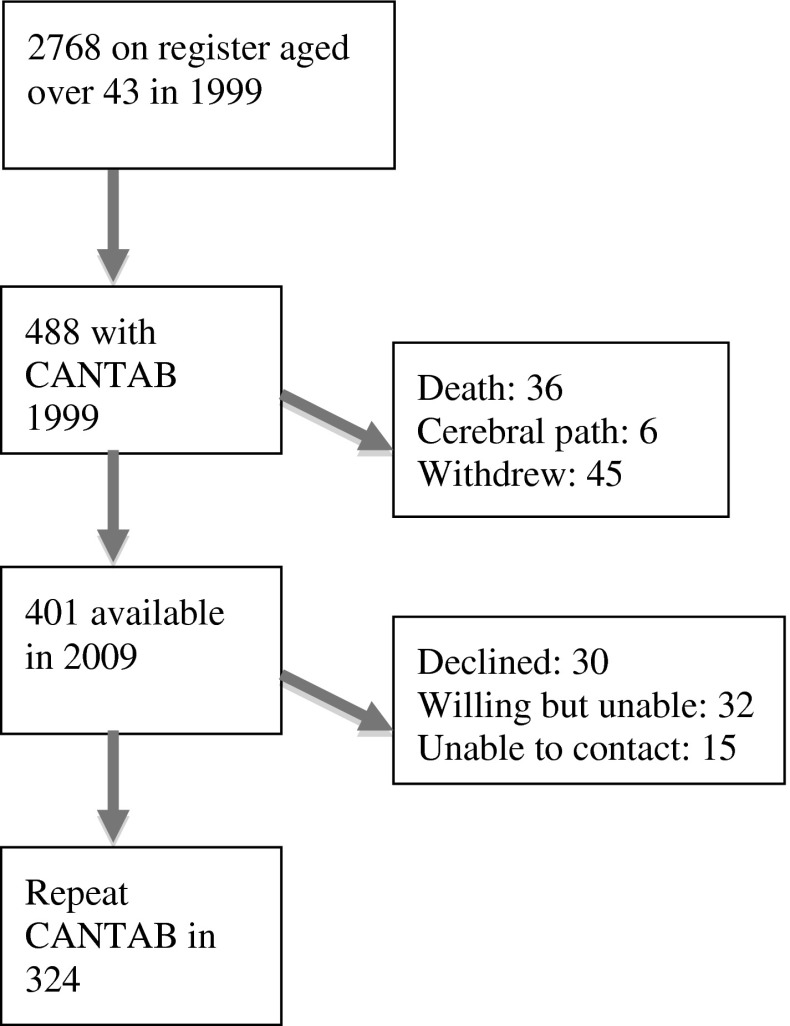
Study participants

In 1999 there were a large number of individuals (2,768) over the age of 43 on the register, (this lower age cut off was arbitrarily chosen to capture the greatest numbers). During 1999, all twins visiting the unit were asked to complete the Cambridge Automated Neuropsychological Test battery (CANTAB). There were no exclusion or selection criteria at this stage. In total, 488 individuals (all women) underwent the CANTAB battery as part of their study visit and their characteristics, compared to the cohort as a whole, are shown in Table 1 and discussed below. The aim of this study was to focus on ‘normal’ ageing and so predefined exclusion criteria for the follow-up study in 2009 were (i) death of one twin and (ii) significant cerebral pathology in one twin (iii) withdrawal from the study. In 2009, 401 of these met study entry criteria (Fig. 1). They were invited back for repeat testing. 324 (127 MZ, 197 DZ) were tested, constituting a 66 % follow-up rate. Zygosity was assessed by use of single nucleotide polymorphism arrays.

**Table 1:** Occupational classification, age and verbal IQ: comparison with general population and whole cohort

	2010 Women on ONS^a^	1999 Whole cohort (%)	1999 Cantab (%) (n = 488)	2009 Cantab (%) (n = 324)	Lost to follow-up (%) (n = 164)
I Professional	13.1	2.9	1.0*	1.6	0
II Intermediate	27.7	26.6	27.8	29.6	25.0
III N Skilled non-manual	45.0	53.3	51.2	50.8	51.0
III M Skilled manual	3.5	11.8	14.8*	15.9*	13.4
IV Partly Skilled	–	3.4	3.4	1.6	6.7***
V Unskilled	10.7	1.9	1.7	0.5	3.9***
Age on 1.1.99 (SD) range	–	56.3 (8.3) 42–79	55.5 (7.8)** 42–72	55.4 (7.4)** 42–72	56.0 (8.5) 42–79
NART	–	–	114.4 (7.5)	114.8 (7.3)	113.7 (7.8)
%male	–	3.8	0	0	0

*NART* National Adult Reading Test, an estimate of verbal IQ stable to ageing and pathology

* *Z* test significantly different from 1999 whole cohort, *p* < 0.05

** *T* test *p* < 0.05 compared to 1999 whole cohort

*** *Z* test “Lost to follow-up” significantly different from 2009 study population, *p* < 0.05

^a^http://www.ons.gov.uk/ons/datasets-andtables/index.html?&newquery=EMP08&content-type=Reference+table&contenttype=Dataset&content-type-orig=%22Dataset%22+OR+contenttype_original%3A%22Reference+table%22

Between 1993 and 1999 all participants in the cohort were asked to complete two separate occupational questionnaires, both of which scored their occupations during their life using the British Registrar General’s scale. Where individuals changed occupation, their paid occupation of longest duration was chosen. Where individuals have answered both questionnaires, the more recent is used (Table 1).

Table 1 also reports the proportions of employed women in each occupational group in 2010. While there are differences in age, cohort and employment classification between our cohort and the general population, these data show that our cohort of volunteers consists mainly of the middling occupations, and under represents both professional and unskilled occupation categories. However, those in this study, and those unselected and lost to follow-up, are broadly similar.

With regard to age, the means for those selected in 1999 and the unselected are close. There was no significant difference in age between those followed up in 2009 and those lost to follow-up. The National Adult Reading Test (NART), an estimate of verbal IQ, was 114 in the 1999 study population and not statistically significantly different in those lost to follow-up.

### Study visits

Informed written consent was obtained for the study which was named ‘Hearing, Learning and Memory’, in an attempt to minimise the impact of stereotypes about ageing (McDaniel et al. 2007). In both visits, twins underwent separate, contemporaneous testing. The NART (24) and CANTAB battery were delivered in the same order (as detailed below), using the same script in 1999 and 2009. Participants were asked to refrain from caffeine for 2 h and alcohol for 12 h before the tests, to continue regular medications, but avoid as-required sedatives for 2 days before the tests. In 1999 additional measures of physical function were also collected as well as detailed questionnaire data. In 2009, the Mini Mental State Examination (MMSE) (Folstein et al. 1975), Geriatric Depression Scale (GDS) (Yesavage et al. 1982), self-reported quality of life (WHOQOL-Bref) (The WHOQOL Group 1994) among other self-reported questionnaires were collected. Both the 1999 and the 2009 studies were conducted in the same institution, with the exception that in 2009 in order for physically frailer individuals to still be included in the study, 14 individuals were tested in their own homes, situated throughout the UK.

### Cognitive measures: CANTAB battery

The CANTAB is a series of tests completed on a touch sensitive screen, and using a response button, which has been standardised using a large elderly population (Robbins et al. 1994). The advantages of using this battery are: (i) that it is largely automated and the scripts given to the operator are standardised, thereby reducing operator influence on performance; (ii) it includes multiple measures of processing speed sensitive to age (Der and Deary 2006; Schaie et al. 2004; Verhaeghen and Salthouse 1997); (iii) while it is “computerised,” it was originally developed in the 1980s and its operation is very basic, requiring no knowledge or familiarity with computers; (iv) each test includes several parallel trials which should increase robustness (Salthouse 2012). However, it contains no verbal cognitive measures. Brief descriptions follow but these tests have been described in detail (Robbins et al. 1994).

### Motor screening

This is a training procedure to relax the subject and introduce them to the equipment. It screens for problems with vision, motor function and comprehension. A series of crosses appear on the screen in different positions and the subject is asked to touch them.

### Pattern recognition memory (PRM)

The subject is asked to remember simple patterns presented one at a time. They are then presented with the familiar pattern and a colour matched pattern of similar complexity. They are asked, “touch the one which you saw before”. The task involves elementary comparison of only two choices, and the accuracy rate is high (87 % in our sample), so this task gives a measure best described as inspection time. Outcome measure: mean latency of a correct response over 24 trials (lowest number of correct trials was 14 in our sample).

### Delayed matching to sample (DMS)

The subject is shown a complex visual pattern made of four coloured sub-elements, and, after a brief delay, four other patterns, one of which is the correct one. She is instructed, “touch the one you saw before”. Again, no suggestion is given to the subject that speed is the measure of interest. There are three practice trials, after which no further instruction is given. In this test the patterns are moderately complex and there are multiple choices and varying delays. The accuracy rate is high, at 84 % in our sample. For these reasons, it is best described as a measure of decision time. Outcome measure: mean correct latency over 40 trials (lowest number of correct trials was 23 in our sample).

### Paired-associates learning (PAL)

This is an episodic memory task. The subject is presented with up to eight individual shapes within eight boxes differently located on the screen. She is then asked to locate the box corresponding to each shape, presented one after the other. Until all responses are correct, the locations are shown again and the subject asked to repeat the location. Outcome measure: number of errors made, adjusted if the participants could only correctly match fewer than eight boxes.

### Spatial span (SSP)

This is a computerised version of the Corsi Blocks task assessing spatial working memory capacity. White boxes on the screen change colour in a sequence. The subject is asked to repeat the sequence back. Outcome measure: longest sequence remembered correctly, given up to three attempts.

### Spatial working memory (SWM)

The test begins with a number of coloured squares (boxes) on the screen. The subject searches for blue tokens, which are hidden one at a time, behind the boxes. She must not revisit previously filled boxes. The aim of this test is that, by a process of elimination, the subject should find one blue ‘token’ in each of the boxes and use them to fill up an empty column at the side. If the subject completes the trial, finding all tokens, the number of boxes increases to a maximum of eight. The first three-box trial is used to explain the test and is not included in the measure. This tests the subject’s ability to retain spatial information and to manipulate remembered items in working memory. It is a self-ordered task and also assesses heuristic strategy and is considered to measure frontal lobe and ‘executive’ function (Owen et al. 1990). Outcome measure: number of ‘between’ errors made (boxes opened which previously contained a token.)

### Reaction time (simple (RTIS) and five-choice (RTIFC))

This measures the speed of response to a visual target (a yellow spot appearing on the screen). The subject places her finger on a button (press pad) and is asked to touch the target on the screen with the same finger, as fast as she can. She is instructed, “Don’t let go of the button until you see the spot.” The measure is taken between stimulus presentation and the moment her finger leaves the press pad, thus eliminating most of the movement time involved. Trials are aborted and repeated if the subject removes their finger before the presentation of the target. Targets are presented with varying pre-stimulus duration. There are practice stages containing a minimum of 30 trials. Outcome measures: simple reaction time (RTIS), where the stimulus is predictable in the centre of the screen (mean of 8 correct trials), and five-choice reaction time (RTIFC), where the stimulus could be in one of five evenly spaced locations (mean of 8 correct trials).

### Analysis and statistical methods

Two types of analysis were conducted, cross sectional analysis at each time point, and analysis of change. The Stata 11 statistical package was used for all transformations, factor analysis and regression modelling (StataCorp 2009).

For the cross sectional analysis at each time point, raw scores were transformed as appropriate. Table 2 gives descriptive statistics of the raw and transformed scores. In addition, a general battery measure was produced using the first un-rotated factor from exploratory factor analysis of all seven transformed measures at each time point separately. This method is frequently used to determine a general factor ‘g’ (Harris and Deary 2011). These cross-sectional first factors captured 75 and 76 % of the shared variance in 1999 and 2009 respectively. Standard univariate structural equation modelling of the contribution of genetic effects and environments to variability in these data was carried out using Mx opensource software (Neale 1994). The stability of these effects over time was then analysed using a bivariate Cholesky decomposition.

**Table 2:** Descriptive statistics of raw test scores

	Mean (SD) 2009 1999	Skewness 2009 1999	Kurtosis 2009 1999	Transform	Transformed mean (SD) 2009 1999	Tranformed skewness 2009 1999	Transformed kurtosis 2009 1999
PAL (errors)	22.2 (22.9)	2.8	11.6	Square root	4.28 (1.97)	1.3	5.7
	21.6 (19.8)	3.2	16.1		4.31 (1.73)	1.3	6.5
DMS (ms)	3664 (1107)	1.5	6.6	Log	8.17 (0.277)	0.4	3.4
	3930 (1254)	1.0	4.2		8.23 (0.312)	-0.3	3.2
PRM (ms)	2227 (571)	1.4	5.6	Log	7.68 (0.236)	0.6	3.5
	2136 (534)	2.0	13.0		7.64 (0.227)	0.5	4.0
SSP (span)	5.23 (1.10)	0.3	3.9	None		–	–
	5.43 (1.13)	0.4	3.8				
SWM (errors)	35.6 (19.3)	0.1	2.4	None		–	–
	33.4 (18.8)	0.2	2.5				
RTIS (ms)	302 (54.6)	2.8	20.1	Log	5.70 (0.159)	1.2	7.1
	365 (75.2)	1.4	5.8		5.88 (0.189)	0.8	3.7
RTIFC (ms)	353 (52.4)	0.5	2.9	Log	5.86 (0.146)	0.2	2.7
	370 (54.5)	1.0	4.4		5.90 (0.140)	0.6	3.4

For analysis of change, we first conducted longitudinal analysis of the change in each cognitive measure. As is universally the case in follow-up studies, there was a significant relationship between raw difference score and baseline score consistent with regression to the mean (Barnett et al. 2005), and mainly for this reason the reliability of raw difference scores are low. This effect is alleviated by using ANCOVA to define change, as is routinely used in the analysis of clinical trial data (Twisk and Proper 2004). For each cognitive measure we used the raw difference scores adjusted for baseline by fitting the baseline test result as a linear covariate. This gives a measure indicating the change in score over 10 years assuming all individuals started at the same baseline performance. It is mathematically the same as fitting the baseline as a covariate with the follow-up measurement as the outcome variable (Twisk 2003), which is the definition of cognitive ageing used by others (Deary et al. 2012). For all tests a positive change score translates as ‘improvement’ over 10 years, and a negative score is decline (scores for SSP have been reversed to allow this) (Table 3).

**Table 3:** Descriptive statistics of change scores

Baseline adjusted change scores^a^	Mean	Median	SD	Skewness	Kurtosis
Change in PAL^b^ (errors)	−1.52e−09	0.189	1.45	−1.2	6.1
Change in DMS (ms)	1.87e−06	152	940	−1.3	7.2
Change in PRM (ms)	−8.80e−07	70.7	493	−1.5	7.4
Change in SSP (span)	2.45e−09	−0.13	0.944	0.1	−3.9
Change in SWM (errors)	−2.28e−10	0.003	14.1	−0.2	3.2
Change in RTIS (ms)	1.07e−08	8.29	49.5	−2.7	19.2
Change in RTIFC (ms)	−1.80e−09	6.41	46.7	−0.3	3.0

^a^All change scores are adjusted for baseline score

^b^Difference in the square root of PAL errors adjusted for baseline score

Univariate structural equation models were then fitted to the raw adjusted change scores to determine the contribution of genetic effects and environments to variability in these change measures.

**Fig. 2:**
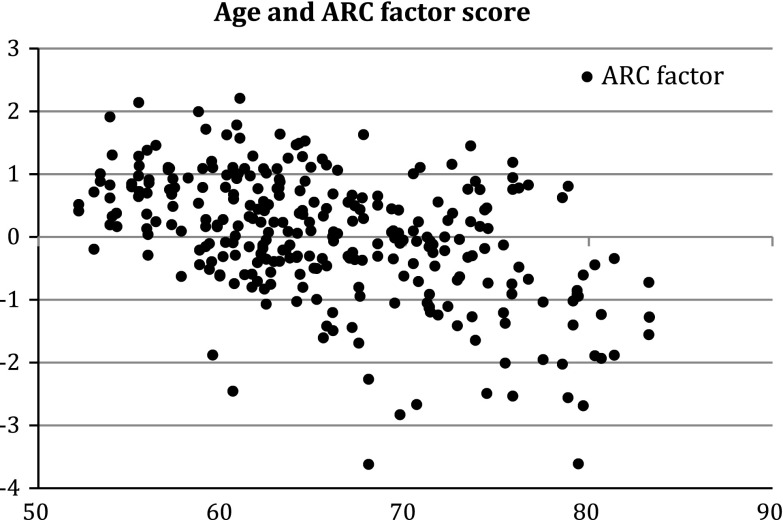
Relationship between Age and ARC factor score. *y-*axis: first principle component of factor analysis of all the change scores adjusted for baseline performance. Scores below 1 indicate declining function over 10 years. *x-*axis: age at second time point

Secondly, in the analysis of change, we attempted to capture change shared between all the tests in the battery. Cross sectional studies show some support for the concept that age-related influence on a wide range of cognitive variables are shared (Verhaeghen and Salthouse 1997). We have further hypothesised that change in cognition over time in the same individual in different tests should have a large common or shared component which is greatest in older individuals. This might be due to an underlying factor or factors, such as degenerative change, which affect change in all the test scores for an individual over time. Indeed, there is significant positive correlation between the change scores, and each is associated with age (Table 4). Using Stata 11, we performed exploratory factor analysis of the seven adjusted delta scores (communalities set to 1, no rotation). This is a similar method to that which is used to identify general cognitive ability at a single time point (Harris and Deary 2011), but the difference is that here we apply it to change scores. To our knowledge (and to the knowledge of Cambridge Cognition, the suppliers of the software), there has been no previous factor modeling applied to change in CANTAB scores. For these reasons, exploratory factor analysis was appropriate and the principal-component factor method in STATA was used. This produced three factors with eigenvalues >1 (top 4 factors 1.76, 1.31, 1.12, and 0.86 respectively). Factor 1 explained 25 % of the variance and is strongly associated with age (standardized beta −0.066 *p* < 0.001, scatter plot against age is shown in Fig. 2). This factor is referred to as the age-related change (ARC) factor in further analysis. Factors 2 and 3 were not associated with age and explained 19 % and 16 % of the variance respectively. Factor loadings are reported in Table 5, showing that the greatest loadings for the ARC factor are on tests with a speed component. While further rotation produces factors more easily interpretable as reaction time, decision time and working memory (data not shown), these are all equally associated with age and none of them support the heuristic of an age-related change factor.

**Table 4:** Pairwise correlations between change measures

Baseline adjusted change scores	Age association (standardized beta)	Pairwise correlations with adjusted change scores						
		PAL	DMS	PRM	SSP	SWM	RTIS	RTIFC
PAL (errors)	−0.03**	1						
DMS (ms)	−0.04***	0.20***	1					
PRM (ms)	−0.04***	0.07	0.39***	1				
SSP (span)	0.03**	0.15**	0.05	0.08	1			
SWM (errors)	−0.03***	0.18**	0.13*	0.14*	−0.20***	1		
RTIS (ms)	−0.03***	−0.07	0.05	0.11*	−0.02	0.06	1	
RTIFC (ms)	−0.04***	0.11*	0.18**	0.13*	−0.05	0.11*	0.44***	1

* *p* < 0.05; ** *p* < 0.01; *** *p* < 0.001. NB. SSP is the only test where a positive delta score means a decline in function, so its sign here is reversed

**Table 5:** Factor loadings for factor analysis of change scores

Variable^a^	Factor 1	Factor 2	Factor 3	Uniqueness
Change in PAL	0.27	0.49	−0.38	0.54
Change in PRM	0.60	−0.16	0.54	0.31
Change in DMS	0.58	−0.20	0.56	0.30
Change in SWM	0.50	−0.31	−0.33	0.55
Change in SSP	0.37	−0.41	−0.42	0.52
Change in RTIS	0.50	0.67	−0.19	0.26
Change in RTIFC	0.59	0.54	−0.24	0.30

*PALsr* square root of paired associates learning errors, *PRMmcl* mean correct latency of pattern recognition memory (ms), *DMSmcl* mean correct latency of delayed matching to sample (ms), *SWMbe* spatial working memory between errors, *SSP* spatial span, *RTIs* simple reaction time (ms), *RTIfc* five choice reaction time (ms). *Uniqueness* refers to variance unique to each variable

^a^Adjusted for baseline

Finally, using phenotypic information collected on these twins in 1999, we tested whether the ARC was associated with known risk factors for cognitive change (Hendrie et al. 2006): vascular risk factors and physical performance level. For this we used multiple linear regression (generalised estimating equation model, adjusting for family structure (Carlin et al. 2005)).

## Results

### Study population characteristics

The study population was well educated and had relatively high self-reported socio-economic status in 2009 (Table 6). The average age at first testing was 55.7 (SD 7.32 range 43–73), and at the second testing 65.7(see Fig. 2). The average MMSE score at the end of the study was 29, similar to a normal ‘healthy’ population, and a significant minority gave a history of depressive illness (17 %) and current GDS > 5 (7 %). 18 % were on a medication identified as being potentially psychoactive, such as opiate analgesia or antidepressants (Table 6). Such potential confounders were included as covariates in the regression models, but as there were no significant differences between monozygotic and dizygotic twins in these population characteristics, they were not included in the twin modelling.

**Table 6:** 2009 Cohort characteristics

2009 Characteristics	Mean/% yes	SD	MZ mean (SD) n = 127	DZ mean (SD) n = 197
Educated > secondary	63 %		62.8 %	63.3 %
Occupation professional/managerial^a^	37 %		39.5 %	34.9 %
MMSE	29.0	1.15	29.0 (1.18)	29.0 (1.14)
GDS > 5	7.0 %		8.4 %	5.8 %
History of depression	17.7 %		14.3 %	20.1 %
Age 2009	65.7	7.32	66.6 (7.56)	65.8 (7.17)
WHOQOL-Bref (max possible 130)	97.0	9.6	97.7 (10.13)	96.6 (9.16)
NART	115.4	9.55	116.0 (8.47)	115.0 (10.1)
Sleep h/day	8.16	1.03	8.14 (1.02)	8.18 (1.03)
Caffeine mg/day	343	229	360 (281)	332 (189)
Psychoactive medication	18.0 %		16.2 %	19.1 %

*MMSE* mini mental state examination, *GDS* geriatric depression score, *WHOQOL-Bref* measure of quality of life, *NART* National Adult Reading Test. An average cup of brewed coffee contains 90 mg caffeine

^a^Occupational status here was taken from a new questionnaire, which asked participants to categorise “your main occupation throughout most of your life.” Categories were professional/managerial, non-manual/clerical, manual, housewife, student, or none

### Cross sectional heritability estimates

**Fig. 3:**
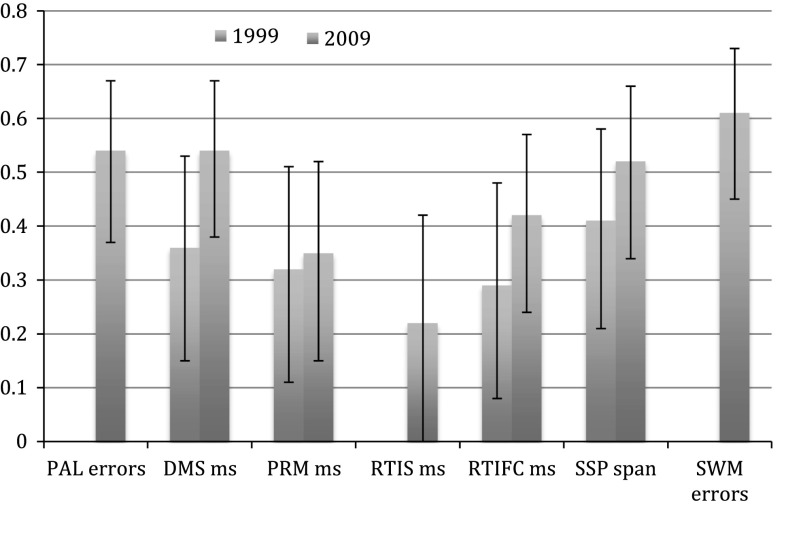
Cross sectional heritability estimates for each cognitive measure. Best fitting model heritability point estimates for each of the tasks and 95 % confidence intervals in the two waves of testing. The best models were CE for PAL errors, RTIS or SWM between errors in 1999

In almost all measures, the heritability estimates slightly increased over the 10 years of follow-up (Fig. 3; Supplementary Table S1). The heritability measures from a general battery factor (first factor, or ‘g’) at each time point were 55 % in 1999, and 76 % in 2009 (Table 7). The factor loadings for the tests were slightly different for 1999 and 2009, especially with regard to DMS and PRM. Measurement invariant scores were computed using the factor loadings from 2009 data. Heritability of these composite measures were almost identical to the estimates in Table 7 (best models AE, with A estimates 75 % (63–83 %) in 2009 and 54 % (36–68 %) in 1999). As is the case in twin studies of many ageing traits, most of the best models were AE models, and the estimated effect of shared environment was minimal. MZ and DZ correlations are found in Supplementary Table S2.

**Table 7:** Heritability of ‘g’ or general cognitive ability factor (first principle component of all transformed measures) calculated in 1999 and 2009

Measure	Model	Estimates % (95 CI)			Model comparison				
		A	C	E	−2LL	χ^2^	df	*p* value	AIC
‘g’ 1999	ACE	48 (0-68)	6 (0-43)	46 (32-68)	843		300		
	**AE**	**55 (37-68)**	**–**	**45 (32-63)**	**843**	**0.09**	**301**	**0.076**	**-1.910**
	CE	–	0.37 (0.23-0.50)	63 (50-77)	847	3.30	301	0.069	1.301
	E	–	–	100	869	26.0	302	0.000	21.999
‘g’ 2009	ACE	76 (42-84)	0 (0-30)	24 (16-35)	804		300		
	**AE**	**76 (65-84)**	**–**	**24 (16-35)**	**804**	**0.00**	**301**	**incalc**	**-2.0**
	CE	–	52 (40-63)	48 (37-60)	820	16.3	301	0.000	14.31
	E	–	–	100	869	65.5	302	0.000	61.5

Models show estimates of A, C and E and 95 % confidence intervals. Lines in bold indicate the best fitting model. −2LL is the −2 times log Likelihood and the χ^2^ test is against the saturated ACE model. Chosen models should be not significantly different from the saturated model (high *p* value), The Akaike Information Criterion (AIC) is a compromise of accuracy and complexity and lower values indicate a better model

### Bivariate analysis

Bivariate modelling of test results at both time points for each individual produced estimates for the variance contributions of A, C and E similar to those reported in the univariate analysis, again showing marginally higher heritabilities at the later time point (see Supplementary Table S3). For the individual measures, models equating these estimates did not have significantly worse fit compared to the saturated models, but the trend over all of them is notable. In the case of the general battery measure, equating the estimates for the two time points trended towards a worse fit (*p* = 0.078). The correlation (r) of the genetic factors contributing to variance across time was very high (0.92–1.0) in all but one of the tests (RTIS) indicating that the genetic factors were largely the same over the 10-year period. Interestingly, E, which includes environmental factors unique to that individual and measurement error, was also, in all cases, significantly correlated between the time points, indicating substantial stability in the environmental factors underpinning variance in cognitive performance over the 10-year period as well. This correlation is strongest in the episodic memory measure PAL (45 %).

### Longitudinal study results

The heritability estimates for change in cognition over time (absolute differences in scores adjusted for baseline performance) were expectedly lower than the cross sectional estimates, but there was still significant effect of a genetic factor seen in 5 of the 7 tests (Table 8). The age related change factor computed from all the change scores together was significantly heritable at 47 % (4–62 %), but in the other two factors heritability estimates did not reach significance. While we do not feel age adjustment is appropriate, because relevant data associated with age would be discarded (see “Discussion and conclusion”), for completeness we have supplied age-adjusted data for the three factors underlying the change scores in Supplementary Table S4, where the heritability of the age-related change factor is reduced to 20 % (0–41 %). Likewise, estimates of heritability for rotated factor solutions were lower: 13 % (0–46 %) for reaction time, 36 %(0–57 %) for decision time, 13 % (0–55 %) for working memory (Supplementary Table S5).

**Table 8:** Heritability of change measures

Measure	Model	Estimates % (95 CI)			Model comparison			
		A	C	E	χ^2^	df	*p* value	AIC
Change in PAL errors^a^	ACE	21 (0–40)	0 (0–24)	79 (60–99)		283		
	**AE**	**21 (1–40)**	**–**	**79 (60–99)**	**0.00**	**284**	**incalc**	**−2.00**
	CE	–	13 (0–28)	87 (72–100)	1.81	284	0.178	−0.19
	E	–	–	100	4.20	285	0.122	0.202
Change in RTIFC (ms)^a^	ACE	17 (0–42)	5 (0–32)	78 (58–98)		278		
	**AE**	**23 (2–42)**	**–**	**77 (58–98)**	**0.04**	**279**	**0.849**	**−1.96**
	CE	–	18 (1–33)	82 (67–99)	0.28	279	0.597	−1.72
	E	–	–	100	4.68	280	0.096	0.68
Change in SSP (span)^a^	ACE	41 (0–58)	0 (0–31)	59 (42–81)		310		
	**AE**	**41 (20–58)**	**–**	**59 (42–80)**	**0.0**	**311**	**incalc**	**−2.0**
	CE	–	25 (10–39)	75 (61–90)	3.32	311	0.068	1.32
	E	–	–	100	13.1	312	0.001	9.31
Change in RTIS (ms)^a^	ACE	27 (0-47)	0 (0-25)	73 (53–97)		300		
	**AE**	**27 (4–47)**	**–**	**73 (53–96)**	**0.0**	**301**	**incalc**	**−2.0**
	CE	–	14 (0–29)	86 (71–100)	1.94	301	0.164	−0.06
	E	–	–	100	5.10	302	0.078	1.10
Change in PRM (ms)^a^	ACE	10 (0–30)	2 (0–26)	88 (70–100)		309		
	AE	12 (0–30)	–	88 (70–100)	0.00	310	0.961	−1.99
	CE	–	10 (0–26)	90 (74–100)	0.09	310	0.770	−1.92
	**E**	**–**	**–**	**100**	**1.58**	**311**	**0.453**	**−2.42**
Change in DMS (ms)^a^	ACE	31 (0–48)	0 (0–24)	69 (52–88)		295		
	**AE**	**31 (12–48)**	**–**	**69 (52–88)**	**0.0**	**296**	**incalc**	**−2.0**
	CE	–	20 (4–35)	80 (65–96)	3.69	296	0.055	1.69
	E	–	–	100	9.64	297	0.008	5.64
Change in SWM (errors)^a^	ACE	11 (0–32)	2 (0–25)	87 (68-100)		307		
	AE	13 (0-32)	–	87 (68–100)	0.01	308	0.933	−1.99
	CE	–	10 (0–25)	90 (75–100)	0.12	308	0.726	−1.88
	**E**	**–**	**–**	**100**	**1.82**	**309**	**0.403**	**−2.18**
ARC Factor	ACE	47 (4–62)	0(0–32)	53 (38–72)		283		
	**AE**	**47 (27–62)**	**–**	**53 (38–73)**	**0.00**	**284**	**incalc**	**−2.0**
	CE	–	31(16–46)	68 (54–84)	4.42	284	0.036	2.42
	E	–	–	100	19.5	285	<0.001	15.51
Factor 2	ACE	22 (0–43)	0 (0–29)	78 (57–100)		285		
	**AE**	**22 (0–43)**	**–**	**78 (57–100)**	**0.0**	**286**	**incalc**	**−2.0**
	CE	–	14 (0–30)	86 (70–100)	0.41	286	0.520	−1.59
	E	–	–	100	3.50	287	0.174	−0.50
Factor 3	ACE	18 (0–38)	0 (0–26)	82 (62–100)		285		
	**AE**	**18 (0–38)**	**–**	**82 (62–100)**	**0.0**	**286**	**incalc**	**−2.0**
	CE	–	11 (0–27)	89 (73–100)	0.91	286	0.340	−1.09
	E	–	–	100	2.72	287	0.257	−1.28

Models show estimates of A, C and E and 95 % confidence intervals. Lines in bold indicate the best fitting model. The χ^2^ test is against the saturated ACE model. Chosen models should be not significantly different from the saturated model (high *p* value). The Akaike Information Criterion (AIC) is a compromise of accuracy and complexity and lower values indicate a better model

^a^Adjusted for baseline

Using phenotypic information collected on these twins in 1999, we tested whether the ARC was associated with known risk factors for cognitive change (Hendrie et al. 2006): vascular risk factors and physical performance (here indicated by leg power on the Nottingham Power Rig). We found, as well as being significantly negatively associated with age, the ARC factor was also negatively associated with history of diabetes (*p* < 0.001) systolic blood pressure (*p* = 0.018) and leg power (*p* < 0.001) at baseline, despite controlling for age, education and occupation status. However, no significant association was found with body mass index. The ARC factor was strongly positively associated with quality of life (WHOQOL-Bref) at follow-up (*p* = 0.002), again controlling for age, education and occupation status.

In order to check whether the heritability in our population is likely to be biased by the relatively higher socioeconomic mix of the cohort, we conducted ad hoc subgroup analysis. Heritability of the ARC factor was calculated in the 27 twins pairs where both twins classified themselves as professional/managerial in 2009 and in the 78 twin pairs where both classified themselves as non-professional/managerial. Supplementary Table S6 shows that in our sample the non-professional subgroup had higher a heritability of the ARC factor (66 %, 19–82 %).

## Discussion and conclusion

This study uses the same cognitive tests in the same older women, performed under the same protocol 10 years apart, and its main finding was significant heritability of change in cognition over the 10 years of the study in 5 of the 7 tests. Moreover, 47 % of the variance in the age-related change factor was found to be attributable to genetic factors. This factor was also predicted by history of diabetes, systolic blood pressure and physical fitness. This lends face validity to this factor as a measure of important cognitive change, and echoes other literature showing the importance of vascular risk factors and physical activity in contributing to longitudinal cognitive change (Hendrie et al. 2006).

Estimates for the heritability of each measure were all marginally higher at the second time point. While for each individual measure models equating estimates for the time points were not significantly different, for the general measure (first factor) such a model trended towards a worse fit, and the consistent trend over all of the tests is apparent, suggesting a definitive difference would have been seen with a larger sample size. The factor loadings of the general measure were slightly different so their definition was not strictly consistent. Computation of scores for each time point based on the factor loadings in 2009 yielded almost identical heritability estimates, indicating that any difference in heritability of ‘g’ is not likely to be due to measurement non-invariance between the two time points.

Heritability of change in variables showing increasing heritability with age is found in some other organ systems in ageing studies, for example motor and lung function (Finkel et al. 2003). Increasing heritability of cognitive function with age would be in keeping with early studies and those focusing on the age range from adolescence into adulthood (Deary et al. 2009), but contrary to more recent studies focusing on older age groups (Lee et al. 2010), and may reflect the relatively young age range of this cohort. Of course, factors differing between the two time points include age, but also include any other development happening within the cohort in this time period.

Heritability of change in cognition is contrary to what has been found in several other twin studies. The Longitudinal Study of Aging Danish Twins (LSADT) showed repeatedly substantial heritability for the intercept parameter and no significant heritability for the slope, testing in waves every 2 years for up to 10 years (McGue and Christensen 2002, 2007). SATSA followed 798 non-demented individuals (including 268 complete twin pairs with two or more time points) in four waves spanning over 13 years, measuring WAIS-R subtests and a standard Swedish ability battery. Numerous studies of this dataset have consistently shown genetic influences on the level of cognitive functioning but only slight, or insignificant influence on the linear slope of change. For example, the heritability of the first principal component measure of general cognitive ability from the SATSA was estimated to be 91 % for the intercept, but only 1 % for the slope (Reynolds et al. 2005). The lack of finding for heritability for linear change has been noted to be an unexpected failure (McGue and Johnson 2007). However, Reynolds’ study did find substantial heritability for quadratic change ranging from 33 to 75 % for non-verbal tests. Our study with only two time points cannot measure quadratic change, but the finding of heritability of the first factor of change in all tests (ARC) in the current study provides some replication for this finding, in that this measure itself changes with age. There is evidence that accelerating change may be more important in predicting cognitive morbidity and mortality (Small and Backman 2007). Indeed accelerating memory decline was seen to be greater in non-demented individuals with specific genetic risk for dementia (TOMM40 L and APOE e4) (Caselli et al. 2012).

There is good evidence that studying speed is of value in cognitive ageing. Age related variance in a range of cognitive variables has been noted to be shared with speed, showing in particular, a close relationship between speed, working memory and fluid intelligence (Verhaeghen and Salthouse 1997). Choice reaction time was found to be significantly associated with mortality in the same sample followed up to age 70, and a stronger predictor of death than general intelligence (Deary and Der 2005a, b). Salthouse contended that age-related increases in variability, which are classically found in computerized tests of speed, are not seen in paper and pencil tests (Salthouse 2000).

There appear to be some differences in the quantitative genetics of speed and working memory measures compared to other cognitive measures. Cross sectional data shows that, contrary to other cognitive measures in advanced age, speed measures continue to increase in heritability from 70 to 80 years of age (Lee et al. 2010). The Swedish Adoption Twin Study of Aging (SATSA) longitudinal study shows heritability of change in some cognitive domains, specifically block design, card rotations and digit span (Reynolds et al. 2005). The WWII veterans’ twin study in the United States has shown that over 9–13 years there is substantial heritability of decline in perceptual speed (48 % at 13 years), but not over 4 years (Lessov-Schlaggar et al. 2007). These studies used measures of perceptual speed using paper and pencil—most commonly the Digit Symbol Substitution Test (DSST) a timed code substitution test.

The CANTAB battery used in the present study was rich in speed measures, and reports their heritability. Interestingly, individual speed measures (and rotated factors separating speed components), were not as heritable as the ARC factor. This would suggest that the heritability for the ARC in the present study is either not related to speed, or related to a speed element which is shared between reaction time, decision time and working memory.

There are some limitations to this study. There were only two time-points, which limits analysis of change, but the interval between testing is likely to be long enough to avoid significant learning effect. The cohort in this study range from mid-life to early old age, so findings reflect developments in late adulthood or the “third age” (Baltes 1997). In later old age, the relative contribution of genetic factors to variation in cognitive ageing may decrease (McArdle and Plassman 2009). The possibility of pre-clinical protopathic bias was minimised by visiting frailer participants at follow-up, and the response rates overall were good.

The study population was volunteer women only, who are generally educated and mainly represent the middling occupations (intermediate and skilled workers). The study sample was unselected from within the whole cohort population and its demographics are similar to the cohort as a whole. In terms of the implications of the socio-economic status of the cohort for heritability estimates, there has been work in younger individuals suggesting a gene*environment interaction between socio-economic status and genetic factors, (such that there is higher heritability of cognitive abilities in more educated and higher socio-economic groups). However, significant recent other work contradicts this (Hanscombe et al. 2012). The only study of which we are aware in adult women suggests no moderating effect of additive genetic effects on cognition in different socio-economic backgrounds (van der Sluis et al. 2008). Indeed, subgroup analysis of our study, found that the lower socioeconomic group (non-professional/managerial) had higher heritability of the ARC factor than the professional group. This would suggest that any bias introduced by having a cohort of relatively higher socioeconomic status would lead to our under-estimating heritability of age-related change. Nevertheless, the domain of this study is older volunteering women, and further studies would be needed to ascertain whether the findings also apply to less educated and unskilled groups, and in particular, to men.

CANTAB testing is very basic to perform, having been devised in the 1980s. It does not require any computer/technology knowledge, so we did not anticipate that recent computer use would give a significant operative advantage. We did not, however, prospectively ask the subjects about their computer use/familiarity, so are unable to test whether variation in experience/familiarity with computers could, to some extent, drive the measured change. The CANTAB battery did not include verbal measures, so the general battery factors created will not necessarily be synonymous with other measures of general cognitive ability (Deary et al. 2009). However, there are some advantages to not including verbal or arithmetic measures. Firstly, there is a body of literature suggesting that speed measures using verbal, lexical or arithmetic information may be less sensitive to the effects of age than speed measures using non-verbal, non-lexical measures (as in this battery) (Salthouse 2000). Secondly, the effects of education/occupation may be reduced in non-verbal as compared with verbal/numerical measures.

We did not feel adjustment for baseline age was appropriate in this case. There was a significant relationship between the change scores and age, but controlling for this would entail discarding change associated with age, and this is specifically of interest to this study (time is part of the causal chain mediating change in cognition). Age does however constitute a shared environment of twins, and so can lead to overestimating the effect of shared environment. In this case, C was never found to be significant and Supplementary Table S4 shows that age adjustment does not significantly alter the estimates of C.

In conclusion, we found substantial heritability of age related change in cognition. This constitutes the second paper reporting that genetic factors may have more influence on accelerating change, as opposed to linear change. Importantly this sort of change, which may be more predictive of subsequent morbidity, may be a better phenotype in the search for genetic pathways involved in cognitive ageing.

## Electronic supplementary material

Below is the link to the electronic supplementary material.

Supplementary material 1 (DOCX 66 kb)
