# Supplementary material for: Cognitive Change in Older Women Using a Computerised Battery: A Longitudinal Quantitative Genetic Twin Study
Source: Behav Genet. 2013 Aug 30;43(6):468–79. doi: 10.1007/s10519-013-9612-z (PMC3825151; doi:10.1007/s10519-013-9612-z)
Supplement: Supplementary file 1 — Supplementary material 1 (DOCX 66 kb) [file 10519_2013_9612_MOESM1_ESM.docx]

Supplementary Data

**Table S1: Results of univariate variance modelling for cognitive variables in 1999 and 2009.**

| **Measure** | **Model** | **Estimates % (95 CI)** | | | **Model Comparison** | | | |
| --- | --- | --- | --- | --- | --- | --- | --- | --- |
|  |  | **A** | **C** | **E** | **χ^2^** | **df** | **pvalue** | **AIC** |
| **PAL 2009** | ACE | 46 (0-66) | 7 (0-43) | 47 (34-67) |  | 317 |  |  |
|  | **AE** | **54 (37-67)** | **-** | **46 (33-63)** | **0.12** | **318** | **0.734** | **-1.885** |
|  | CE | - | 39(25-51) | 61 (49-75) | 3.43 | 318 | 0.064 | 1.431 |
|  | E | - | - | 100 | 29.6 | 319 | 0.000 | 25.624 |
| **PAL 1999** | ACE | 22 (0-57) | 16 (0-42) | 62 (43-84) |  | 308 |  |  |
|  | AE | 42 (22-59) | - | 58 (41-78) | 0.57 | 309 | 0.450 | -1.430 |
|  | **CE** | **-** | **30 (15-43)** | **70 (57-85)** | **0.51** | **309** | **0.474** | **-1.488** |
|  | E | - | - | 100 | 14.8 | 310 | 0.001 | 10.774 |
| **DMS 2009** | ACE | 54 (22-67) | 0 (0-25) | 46 (33-62) |  | 315 |  |  |
|  | **AE** | **54 (38-67)** | **-** | **46 (33-62)** | **0.0** | **316** | **incalc** | **-2.0** |
|  | CE | - | 37 (23-50) | 63 (50-77) | 8.30 | 316 | 0.004 | 6.296 |
|  | E | - | - | 100 | 32.5 | 317 | 0.000 | 28.497 |
| **DMS 1999** | ACE | 36 (0-53) | 0 (0-24) | 64 (47-85) |  | 309 |  |  |
|  | **AE** | **36 (15-53)** | **-** | **64 (47-85)** | **0.00** | **310** | **incalc** | **-2.0** |
|  | CE | - | 22 (6-36) | 78 (64-94) | 3.9 | 310 | 0.048 | 1.900 |
|  | E | - | - | 100 | 11.4 | 311 | 0.003 | 7.397 |
| **PRM 2009** | ACE | 35 (0-52) | 0 (0-27) | 65 (48-84) |  | 315 |  |  |
|  | **AE** | **35 (16-52)** | **-** | **65 (48-84)** | **0.0** | **316** | **incalc** | **-2.0** |
|  | CE | - | 23 (8-37) | 77 (63-92) | 3.50 | 316 | 0.062 | 1.495 |
|  | E | - | - | 100 | 12.0 | 317 | 0.002 | 7.991 |
| **PRM 1999** | ACE | 32 (0-51) | 0 (0-25) | 68 (49-89) |  | 309 |  |  |
|  | **AE** | **32 (11-51)** | **-** | **68 (49-89)** | **0.0** | **310** | **incalc** | **-2.0** |
|  | CE | - | 19 (3-33) | 81 (67-97) | 3.16 | 310 | 0.075 | 1.165 |
|  | E | - | - | 100 | 8.65 | 311 | 0.013 | 4.65 |
| **SSP 2009** | ACE | 52 (4-66) | 0 (0-35) | 48 (34-68) |  | 317 |  |  |
|  | **AE** | **52 (34-66)** | **-** | **48 (34-66)** | **0** | **318** | **incalc** | **-2.0** |
|  | CE | - | 35 (20-48) | 65 (52-80) | 4.47 | 318 | 0.035 | 2.469 |
|  | E | - | - | 100 | 37.2 | 249 | 0.000 | 33.26 |
| **SSP 1999** | ACE | 37 (0-58) | 4 (0-38) | 60 (42-84) |  | 310 |  |  |
|  | **AE** | **41 (21-58)** | **-** | **59 (42-79)** | **0.03** | **311** | **0.866** | **-1.971** |
|  | CE | - | 28 (12-41) | 72 (59-88) | 1.5 | 311 | 0.218 | -0.479 |
|  | E | - | - | 100 | 13.9 | 312 | 0.001 | 9.876 |
| **SWM 2009** | ACE | 52 (6-72) | 7 (0-41) | 40 (28-60) |  | 317 |  |  |
|  | **AE** | **61 (45-73)** | **-** | **39 (27-55)** | **0.17** | **318** | **0.68** | **-1.831** |
|  | CE | - | 41 (28-53) | 59 (47-72) | 4.70 | 318 | 0.03 | 2.699 |
|  | E | - | - | 100 | 34.6 | 319 | 0.000 | 30.626 |
| **SWM 1999** | ACE | 15 (0-54) | 21 (0-44) | 64 (46-83) |  | 307 |  |  |
|  | AE | 40 (21-56) | - | 60 (44-79) | 0.92 | 308 | 0.338 | -1.083 |
|  | **CE** | **-** | **31 (16-45)** | **69 (55-84)** | **0.28** | **308** | **0.595** | **-1.717** |
|  | E | - | - | 100 | 15.9 | 309 | 0.000 | 11.916 |
| **RTIS 2009** | ACE | 16 (0-42) | 4 (0-29) | 80 (58-100) |  | 300 |  |  |
|  | **AE** | **22 (0-42)** | **-** | **78 (58-100)** | **0.04** | **301** | **0.85** | **-1.965** |
|  | CE | - | 15 (0-30) | 85 (70-100) | 0.22 | 301 | 0.64 | -1.781 |
|  | E | - | - | 100 | 3.54 | 302 | 0.17 | -0.456 |
| **RTIS 1999** | ACE | 2 (0-41) | 17(0-33) | 81 (59-98) |  | 301 |  |  |
|  | AE | 24 (2-43) |  | 76 (57-98) | 0.52 | 302 | 0.47 | -1.477 |
|  | **CE** | **-** | **18 (2-33)** | **82 (67-98)** | **0.00** | **302** | **0.952** | **-1.996** |
|  | E | - | - | 100 | 5.12 | 303 | 0.077 | 1.122 |
| **RTIFC 2009** | ACE | 30 (0-57) | 10 (0-43) | 60 (43-80) |  | 316 |  |  |
|  | **AE** | **42 (24-57)** | **-** | **58 (43-76)** | **0.22** | **317** | **0.64** | **-1.782** |
|  | CE | - | 32 (18-45) | 68 (55-82) | 1.25 | 317 | 0.264 | -0.751 |
|  | E | - | - | 100 | 18.9 | 318 | 0.000 | -14.919 |
| **RTIFC 1999** | ACE | 29 (0-48) | 0 (0-33) | 71 (52-94) |  | 301 |  |  |
|  | **AE** | **29 (8-48)** | **-** | **71 (52-92)** | **0.00** | **302** | **incalc** | **-2.0** |
|  | CE | - | 20 (4-34) | 80 (66-96) | 0.87 | 302 | 0.352 | -1.132 |
|  | E | - | - | 100 | 6.95 | 303 | 0.031 | 2.945 |

Models show estimates of A, C and E and 95% confidence intervals. Lines in bold indicate the best fitting model. The cross sectional raw data were transformed prior to modelling to ensure near normal distribution as follows: PAL (errors), square root; DMS, PRM, RTIS, RTIFC (all ms), log transform. No transformation was required for SSP (span) or SWM (errors).

**Table S2 : Correlations in MZ and DZ twins for all variables.**

|  | **MZ**  **Corr. 1999** | **DZ**  **Corr. 1999** | **MZ**  **Corr. 2009** | **DZ**  **Corr. 2009** |  | **MZ Corr.** | **DZ Corr.** |
| --- | --- | --- | --- | --- | --- | --- | --- |
| **PAL** | 0.30 ^b^ | 0.30 ^b^ | 0.57 ^b^ | 0.30 ^b^ | **Change in PAL^a^** | 0.38 ^b^ | -0.01 |
| **DMS** | 0.42 ^b^ | 0.06 | 0.63 ^b^ | 0.15 | **Change in DMS^a^** | 0.44 ^b^ | -0.03 |
| **PRM** | 0.38 ^b^ | 0.06 | 0.42 | 0.09 | **Change in PRM^a^** | 0.14 | 0.05 |
| **SSP** | 0.35 ^b^ | 0.24 ^b^ | 0.51 ^b^ | 0.26 ^b^ | **Change in SSP^a^** | 0.40 ^b^ | 0.16 |
| **SWM** | 0.36 ^b^ | 0.29 ^b^ | 0.52 ^b^ | 0.36 ^b^ | **Change in SWM^a^** | 0.15 | 0.07 |
| **RTIS** | 0.18 | 0.19 | 0.18 | 0.13 | **Change in RTIS^a^** | 0.30 ^b^ | 0.07 |
| **RTIFC** | 0.27 ^b^ | 0.16 | 0.44 ^b^ | 0.24 ^b^ | **Change in RTIFC^a^** | 0.24 | 0.12 |
|  | | | | | **ARC Factor** | 0.51 ^b^ | 0.17 |
|  |  |  |  |  | **Factor 2** | 0.19 | 0.12 |
|  |  |  |  |  | **Factor 3** | 0.23 | 0.02 |

The cross sectional raw data were transformed to ensure near normal distribution as follows: PAL (errors), square root; DMS, PRM, RTIS, RTIFC (all ms), log transform. No transformation was required for SSP (span) or SWM (errors).

^a^Adjusted for baseline. ^b^Significant correlation (p<0.05)

**Table S3: Results of bivariate variance modelling for cognitive change variables**

|  | **Model fit** | | | **Model comparison** | | **Estimates % (95% CI)**  **First row 1999,**  **second row 2009** | | | **Corr. of estimates across time (95%CI)** | | |
| --- | --- | --- | --- | --- | --- | --- | --- | --- | --- | --- | --- |
|  | **Model** | **-2 LL** | **df** | **p-val** | **AIC** | **A** | **C** | **E** | **rA** | **rC** | **rE** |
| **PAL (errors)** | ACE | 2383.7 | 628 |  |  | 29  (0-58) | 10  (0-38) | 60  (42-81) | 0.92  (-0.99-1.0) | 1.0  (-1-1.0) | 0.47  (0.28-0.63) |
|  |  |  |  |  |  | 46  (1-66) | 7  (0-39) | 47  (34-67) |  |  |  |
|  | **ACE without rC** | **2384.1** | **629** | **0.57** | **-1.7** | **42**  **(19-59)** | **0**  **(0-12)** | **58**  **(41-78)** | **0.92**  **(0.78-1.0)** | **-** | **0.45**  **(0.28-0.60)** |
|  |  |  |  |  |  | **54**  **(33-67)** | **0**  **(0-13)** | **46**  **(33-62)** |  |  |  |
| **DMS (ms)** | ACE | 98.23 | 627 |  |  | 32  (3-49) | 0  (0-25) | 68  (51-86) | 0.94  (0.64-1.0) | 0.99  (-1-1.0) | 0.28  (0.09-0.46) |
|  |  |  |  |  |  | 53  (19-66) | 0  (0-27) | 47  (34-63) |  |  |  |
|  | **ACE without rC** | **98.23** | **628** | **1.0** | **-2.0** | **32**  **(14-49)** | **0**  **(0-10)** | **68**  **(51-86)** | **0.94**  **(0.70-1.0)** | **-** | **0.28**  **(0.09-0.46)** |
|  |  |  |  |  |  | **53**  **(34-66)** | **0**  **(0-12)** | **47**  **(34-63)** |  |  |  |
| **PRM (ms)** | ACE | -194.9 | 627 |  |  | 36  (13-53) | 0  (0-16) | 64  (47-82) | 1  (0.85-1) | 0.78  (-1-1) | 0.29  (11-44) |
|  |  |  |  |  |  | 40  (15-55) | 0  (0-17) | 60  (45-78) |  |  |  |
|  | **ACE without rC** | **-194.9** | **628** | **1.0** | **-2.0** | **36**  **(18-53)** | **0**  **(0-8)** | **64**  **(47-82)** | **1.0**  **(0.85-1.0)** | **-** | **0.29**  **(0.11-0.44)** |
|  |  |  |  |  |  | **40**  **(21-55)** | **0**  **(0-9)** | **60**  **(45-78)** |  |  |  |
| **SSP (span)** | ACE | 1822.0 | 628 |  |  | 20  (0-53) | 12  (0-36) | 68  (47-86) | 1.00  (0.71-1.0) | 1  (-1-1) | 29  (9-50) |
|  |  |  |  |  |  | 48  (0-64) | 2  (0-37) | 50  (36-70) |  |  |  |
|  | **ACE without rC** | **1822.1** | **629** | **0.72** | **-1.9** | **26**  **(11-53)** | **7**  **(0-19)** | **66**  **(47-85)** | **1.0**  **(0.61-1.0)** | **-** | **0.28**  **(0.08-0.47)** |
|  |  |  |  |  |  | **50**  **(21-64)** | **0**  **(0-20)** | **50**  **(36-68)** |  |  |  |
| **SWM**  **(errors)** | ACE | 2393.0 | 627 |  |  | 40  (4-58) | 4  (0-33) | 56  (41-74) | 0.99  (0.78-1.0) | 1  (-1-1) | 0.38  (0.20-0.55) |
|  |  |  |  |  |  | 52  (8-72) | 7  (0-39) | 41  (28-60) |  |  |  |
|  | **ACE without rC** | **2393.1** | **628** | **0.67** | **-1.8** | **44**  **(28-59)** | **0**  **(0-8)** | **56**  **(41-72)** | **0.99**  **(0.87-1.0)** | **-** | **0.37**  **(0.20-0.53)** |
|  |  |  |  |  |  | **60**  **(40-72)** | **0**  **(0-11)** | **40**  **(28-56)** |  |  |  |
| **RTIS**  **(ms)** | ACE | -497.3 | 604 |  |  | 09  (0-41) | 12  (0-32) | 79  (59-96) | 1  (-1-1) | 1  (-1-1) | 0.36  (0.18-0.50) |
|  |  |  |  |  |  | 10  (0-40) | 8  (0-28) | 82  (60-98) |  |  |  |
|  | **ACE without rA** | **-497.1** | **605** | **0.66** | **-1.8** | **0**  **(0-17)** | **18**  **(3-32)** | **82**  **(66-96)** | **-** | **1**  **(0.54-1.0)** | **0.38**  **(0.26-0.50)** |
|  |  |  |  |  |  | **0**  **(0-17)** | **14**  **(2-29)** | **86**  **(68-98)** |  |  |  |
| **RTIFC**  **(ms)** | ACE | -768.6 | 620 |  |  | 25  (0-46) | 2  (0-29) | 23  (54-90) | 1  (-0.23-1.0) | 1  (-1-1) | 0.22  (0.04-0.42) |
|  |  |  |  |  |  | 39  (0-58) | 4  (0-39) | 57  (42-76) |  |  |  |
|  | **ACE without rC** | **-768.6** | **621** | **0.86** | **-2.0** | **27**  **(10-46)** | **0**  **(0-13)** | **73**  **(54-89)** | **1.0**  **(0.70-1.0)** | **-** | **0.22**  **(0.04-0.40)** |
|  |  |  |  |  |  | **43**  **(19-58)** | **0**  **(0-18)** | **57**  **(42-74)** |  |  |  |
| **‘g’** | ACE | 1460.3 | 603 |  |  | 49  (17-67) | 3  (0-31) | 47  (33-64) | 0.98  (0.85-1.0) | 1  (-1-1) | 0.33  (0.11-0.53) |
|  |  |  |  |  |  | 75  (45-84) | 1  (0-27) | 24  (16-35) |  |  |  |
|  | **ACE without rC** | **1460.3** | **604** | **0.86** | **-2.0** | **53**  **(35-67)** | **0**  **(0-11)** | **47**  **(33-64)** | **0.96**  **(0.86-1.0)** | **-** | **0.33**  **(0.11-0.52)** |
|  |  |  |  |  |  | **76**  **(60-84)** | **0**  **(0-11)** | **24**  **(16-35)** |  |  |  |

Results of bivariate structural equation modelling are given for all the phenotypes. The nested models test which of the correlations across time can be removed from the model without significant loss of fit. In all cases except RTIS, removing the rC term resulted in the best models which are depicted in bold. All these nested models assume that ACE is the best fitting model for both phenotypes individually. Estimated variances explained by the specific causal factors are given in two rows, 1999 above and 2009 below. The correlation between A, C and E across time are presented. The cross sectional raw data were transformed prior to modelling to ensure near normal distribution as follows: PAL, square root; DMS, PRM, RTIS, RTIFC, log transform. No transformation was required for SSP or SWM.

**Table S4: Analysis of change factor heritabilities after adjustment for age.**

| **Measure** | **Model** | **Estimates % (95 CI)** | | | **Model Comparison** | | | |
| --- | --- | --- | --- | --- | --- | --- | --- | --- |
|  |  | A | C | E | χ^2^ | df | pvalue | AIC |
| **ARC Factor adjusted for age** | ACE | 20 (0-41) | 0 (0-21) | 80 (59-100) |  | 285 |  |  |
|  | **AE** | **20 (0-41)** | **-** | **80 (59-100)** | **0.0** | **286** | **incalc** | **-2.0** |
|  | CE | - | 9 (0-25) | 91 (75-100) | 1.67 | 286 | 0.196 | -0.33 |
|  | E | - | - | 100 | 2.87 | 287 | 0.238 | -1.13 |
| **Factor 2 adjusted for age** | ACE | 22 (0-43) | 0 (0-29) | 78 (57-100) |  | 285 |  |  |
|  | **AE** | 22 (0-43) | - | 78 (57-100) | **0.0** | **286** | **incalc** | **-2.0** |
|  | CE | - | 14 (0-30) | 86 (70-100) | 0.41 | 286 | 0.523 | -1.59 |
|  | E | - | - | 100 | 3.50 | 287 | 0.174 | -0.50 |
| **Factor 3 adjusted for age** | ACE | 18 (0-38) | 0 (0-25) | 82 (62-100) |  | 285 |  |  |
|  | **AE** | **18 (0-38)** | **-** | **82 (62-100)** | **0.0** | **286** | **Incalc** | **-2.0** |
|  | CE | - | 11 (0-27) | 89 (73-100) | 0.91 | 286 | 0.335 | -1.07 |
|  | E | - | - | 100 | 2.73 | 287 | 0.256 | -1.27 |

**Table S5: ACE estimates for rotated factor solutions**

| **Measure** | **Model** | **Estimates % (95 CI)** | | | **Model Comparison** | | | |
| --- | --- | --- | --- | --- | --- | --- | --- | --- |
|  |  | A | C | E | χ^2^ | df | pvalue | AIC |
| **Rotated f1**  (Change in reaction time (RTIS & RTIFC)) | ACE | 14 (0-46) | 9(0-46) | 77 (54-100) |  |  |  |  |
|  | AE | 23 (0-46) | - | 77 (54-100) | 0.00 | 109 | 1.0 | -2.0 |
|  | CE | - | 23 (0-46) | 77 (54-100) | 0.00 | 109 | 1.0 | -2.0 |
|  | E | - | - | 100 | 3.29 | 110 | 0.19 | -0.7 |
| **Rotated f2**  (Change in decision time (PRM & DMS)) | ACE | 36 (0-57) | 0 (0-57) | 63 (42-88) |  |  |  |  |
|  | AE | 37 (12-57) | - | 63 (42-88) | 0.00 | 109 | incalc | -2.0 |
|  | CE | - | 37 (12-57) | 63 (42-88) | 0.00 | 109 | incalc | -2.0 |
|  | E | - | - | 100 | 8.07 | 110 | 0.018 | 4.08 |
| **Rotated f3**  (Change in working memory (PAL, SWM and SSP)) | ACE | 13 (0-55) | 21 (0-55) | 66 (45-91) |  |  |  |  |
|  | AE | 34 (9-55) | - | 66 (45-91) | 0.0 | 109 | incalc | -2.0 |
|  | CE | - | 34 (9-55) | 66 (45-91) | 0.0 | 109 | incalc | -2.0 |
|  | E | - | - | 100 | 6.7 | 110 | 0.035 | 2.73 |

**Table S6: Subgroup analysis of Age-Related Change Factor by both twins’ occupational status**

| **Measure** | **Model** | **Estimates % (95 CI)** | | | **Model Comparison** | | | |
| --- | --- | --- | --- | --- | --- | --- | --- | --- |
|  |  | A | C | E | χ^2^ | df | pvalue | AIC |
| **ARC factor** where both twins are professional/managerial | ACE | 0 (0-67) | 37 (0-65) | 63 (31-100) |  | 45 |  |  |
|  | AE | 40 (0-72) | - | 60 (28-100) | 1.41 | 46 | 0.236 | -0.60 |
|  | **CE** | **-** | **37 (0-65)** | **63 (35-100)** | **0.0** | **46** | **incalc** | **-2.0** |
|  | E | - | - | 100 | 3.74 | 47 | 0.154 | -0.26 |
| **ARC factor** where both twins are non-professional/managerial | ACE | 66 (19-82) | 0 (0-31) | 34 (18-63) |  | 120 |  |  |
|  | **AE** | **66 (19-82)** | - | 34 (18-63) | **0.0** | **121** | **incalc** | **-2.0** |
|  | CE | - | 37 (14-56) | 63 (44-86) | 5.98 | 121 | 0.015 | 3.98 |
|  | E | - | - | 100 | 15.3 | 122 | 0.000 | 11.31 |

N.B There were only 27 twin pairs where both twins described themselves as professional/ managerial so confidence intervals for this subgroup are wide.
